# A parsimonious nomogram for individualized prediction of 1-year functional outcome after STN-DBS in Parkinson’s disease: a single-center retrospective study

**DOI:** 10.3389/fneur.2026.1779907

**Published:** 2026-02-20

**Authors:** Yiming Zhang, Qiushi Zhu, Baojun Fang, Fengyang Geng

**Affiliations:** Department of Neurosurgery, Liaocheng People’s Hospital, Liaocheng, Shandong, China

**Keywords:** calibration, decision curve analysis, deep brain stimulation, functional outcome, nomogram, Parkinson’s disease, risk prediction, subthalamic nucleus

## Abstract

**Background:**

In the context of Parkinson’s disease (PD), subthalamic nucleus deep brain stimulation (STN-DBS) has been shown to alleviate motor symptoms; nevertheless, functional outcomes at follow-up continue to be inconsistent. Patients who are at a higher risk of unsatisfactory functional recovery could be identified with the use of a personalized risk stratification tool, which would also provide information for perioperative treatment.

**Methods:**

We retrospectively reviewed consecutive PD patients who underwent STN-DBS at the Department of Functional Neurosurgery, Liaocheng People’s Hospital, from January 1, 2015 to August 1, 2024, with 1-year follow-up. The outcome was defined using the medication-off Schwab and England Activities of Daily Living Scale (S&E) at 1 year: good outcome (S&E > 70) versus poor outcome (S&E ≤ 70). Candidate predictors were prespecified and collected from three domains: general clinical characteristics, perioperative indicators, and preoperative specialist assessments. Through the use of multivariable logistic regression, independent predictors of poor outcomes were found, and a nomogram was produced. Bootstrap resampling was utilized in order to carry out the internal validation process. The area under the receiver operating characteristic curve [AUC (C-index)] was used to quantify the discrimination of the model, the calibration was investigated using a calibration plot and the Hosmer–Lemeshow test, and the clinical utility was evaluated using decision curve analysis (DCA).

**Results:**

184 people were included in the study, with 109 having a positive outcome and 75 having a negative outcome. Out of the 195 patients that were eligible, 11 were lost to follow-up. Independently, worse outcome was linked with older age (odds ratio 1.08, 95% confidence interval 1.03–1.14), a lower score on the Mini-Mental State Examination (MMSE) (odds ratio 0.66, 95% confidence interval 0.56–0.79), and postoperative electrolyte disorder (odds ratio 2.97, 95% confidence interval 1.28–6.91). There was a low level of optimism on the internal validation (optimism-corrected C-index 0.841), despite the fact that the nomogram shown decent discrimination (AUC 0.846; bootstrap 95% confidence interval 0.781–0.905). In general, the calibration revealed that the projected risks and the observed hazards were in agreement. The results of the DCA indicated that the model had a positive net benefit across a wide range of threshold probabilities (0.20–0.99).

**Conclusion:**

For the purpose of predicting the functional prognosis of a patient with Parkinson’s disease 1 year after receiving STN-DBS, we constructed a parsimonious nomogram that included age, MMSE, and postoperative electrolyte imbalance. In the event that our model is validated by an external source, it may be able to facilitate individualized perioperative risk assessment and collaborative decision-making.

## 1 Introduction

Parkinson’s disease (PD) is a neurodegenerative disorder prevalent in older adults, characterized by progressive motor impairment (rigidity, bradykinesia, and resting tremor) as well as a wide range of non-motor symptoms such as cognitive decline, sleep disturbances, mood disorders, and autonomic dysfunction. Together, these manifestations pose a substantial threat to daily functioning and quality of life and place a growing burden on families and healthcare systems (1–4).

Even though dopaminergic therapy is the foundation of Parkinson’s disease (PD) management, a significant number of patients continue to have motor fluctuations, dyskinesia, and problems associated to medication over time. Subthalamic nucleus deep brain stimulation (STN-DBS) has become an established intervention for patients who have been carefully selected over the course of the previous two decades as a result of advancements in stereotactic neurosurgery, neuroimaging guiding, and intraoperative electrophysiological mapping. A significant number of patients can see major functional gains as a result of STN-DBS, which can alleviate motor symptoms and reduce the amount of medication that is required (5–7). Nevertheless, a persistent clinical problem continues to exist: postoperative functional recovery is uneven, and a percentage of patients experiences unsatisfactory functional outcomes despite the fact that the treatments were technically effective. The decision-making process for candidates, perioperative planning, and expectation management are all made more difficult by this variability.

From a practical standpoint, clinicians need tools that can translate routinely available information into individualized risk estimates. Existing studies have explored numerous predictors of DBS outcomes, yet several gaps limit bedside applicability. First, many reports emphasize symptom-based scales or group-level averages rather than patient-centered functional independence (8, 9). Second, candidate factors are often evaluated in isolation or embedded within complex models that are difficult to implement in routine workflows. Third, even when discrimination is reported, calibration and—critically—clinical utility are less consistently examined, leaving uncertainty about whether a model would meaningfully improve decision-making (10, 11). Recent machine-learning research has also investigated non-invasive voice-based approaches to predict PD progression and UPDRS (Unified Parkinson’s Disease Rating Scale) scores, further underscoring the demand for individualized prediction tools in Parkinson’s disease (12).

Nomograms offer an interpretable framework for individualized prediction by converting multivariable regression models into a simple scoring system (12, 13). When rigorously validated, they can support risk stratification and facilitate clinician–patient communication. Nevertheless, nomogram-based tools that target functional outcome after STN-DBS and are evaluated not only for discrimination but also for calibration and potential clinical benefit remain limited, particularly in real-world surgical cohorts.

For the purpose of predicting the functional outcome of patients with Parkinson’s disease one year after receiving STN-DBS procedures, we created and internally verified a parsimonious nomogram by using a retrospective real-world cohort that was provided by a high-volume functional neurosurgery clinic. This nomogram was created with the intention of predicting the functional outcome. After a year had passed, we used the Schwab and England Activities of Daily Living Scale (S&E) to evaluate the functional result. This scale was administered after the patient had stopped taking their medication. A score of S&E that was equal to or lower than 70 suggested a less favorable outcome, whereas a score of S&E that was more than 70 indicated a more favorable end with respect to the outcome. Our model takes into account (i) baseline demographics (age), (ii) cognitive status (Mini-Mental State Examination, MMSE), and (iii) an actionable perioperative factor, which is a postoperative electrolyte problem that occurs during hospitalization and may be amenable to prevention and early care. It is important to note that our model incorporates all of these factors. In addition to the building of the model, we carried out bootstrap internal validation, quantified discrimination by utilizing the area under the receiver operating characteristic curve [AUC (C-index)], evaluated calibration, and evaluated clinical utility by means of decision curve analysis (DCA). Our intention is to integrate interpretability, internal validation, and utility-oriented evaluation in order to accomplish our objective of offering a clinically deployable tool for individualized risk assessment and shared decision-making. We also plan to lay the foundation for external validation in multicenter cohorts, which is another one of our goals.

## 2 Materials and methods

### 2.1 Subjects

Participants in the study were eligible to enroll if they were diagnosed with Parkinson’s disease (PD) and had received subthalamic nucleus deep brain stimulation (STN-DBS) at the Department of Functional Neurosurgery, Brain Hospital of Liaocheng People’s Hospital between the dates of January 1, 2015 and August 1, 2024. Patients were followed up for a period of one year, and those who were not able to be followed up on were not included. This study was given the go-ahead by the institutional ethics committee (Approval No. LW20150305), and signed informed permission was obtained from each and every patient.

### 2.2 Outcome measures and definitions

#### 2.2.1 Inclusion criteria

The diagnostic criteria for Parkinson’s disease (PD) were satisfied by each and every patient (14). The age greater than 18 years; The clinical response to levodopa that has been documented; The occurrence of symptomatic motor fluctuations and/or dyskinesia that is resistant to pharmacological control is associated with a diminishing effect from medication. This study was successful in obtaining patients’ written informed consent, and they agreed to take part in the research.

#### 2.2.2 Exclusion criteria

The presence of various malignant neoplasms concurrently with the presence of an intracranial tumor participation in additional clinical trials and research that are currently ongoing.

### 2.3 STN-DBS surgical procedure

STN-DBS was performed using standard stereotactic techniques. Levodopa preparations were withheld for at least 12 h before surgery. Preoperative imaging (CT and/or MRI) was used for target planning based on the AC-PC reference, and intraoperative microelectrode recording and test stimulation were applied when appropriate to refine target localization.

Bilateral STN electrodes were implanted through frontal burr holes and secured, followed by implantation of the pulse generator in a subclavicular pocket with subcutaneous tunneling and connection to the extension cables under general anesthesia. Postoperative imaging was used to confirm lead position. A detailed step-by-step surgical description is provided in Supplementary material S1. For lead-placement verification, a postoperative thin-slice CT was routinely obtained and fused with the preoperative MRI to assess the concordance between the actual electrode location and the planned STN target; in our routine quality-control review, lead placement was consistently satisfactory, with deviations typically within 2 mm and no revision surgery required for malposition.

### 2.4 Observation variables and assessment methods

It was necessary to go through the computerized medical records in order to acquire generic clinical information. This information comprised gender, age, age at which symptoms first appeared, and educational level. Additionally, perioperative parameters were collected, which included the length of time the operation lasted, the volume of blood that was lost during the procedure, and the grading of the anesthetic risk given by the American Society of Anesthesiologists (ASA). The Hoehn and Yahr (H&Y) stage (15), the Unified Parkinson’s Disease Rating Scale (UPDRS) parts II and III (16), the Non-Motor Symptoms Scale (NMSS) (17), the Mini-Mental State Examination (MMSE) (18), the Parkinson’s Disease Sleep Scale (PDSS) (19), the Parkinson’s Disease Questionnaire-39 (PDQ-39) (15), the Beck Anxiety Inventory (BAI) (20), the Beck Depression Inventory (BDI) (13), and the Schwab and England Activities of Daily Living scale (S&E) (21) were some of the assessments that were specific to Parkinson’s disease.

The S&E scale was graded as follows: 100, completely independent; 90, fully independent with mild slowness; 80, independent in most situations but requires approximately twice the normal time to complete daily activities; 70, not completely independent, experiences difficulty in managing daily activities and requires approximately 3–4 times the normal time; 60, partially dependent on others and unable to complete most activities; 50, dependent on others, requiring assistance for approximately half of daily activities; 40, unable to live independently, requiring assistance for most activities; 30, requiring assistance for nearly all activities; 20, able to do a few things but unable to complete any activities of daily living independently; 10, completely dependent and severely disabled; and 0, bedridden with severe autonomic dysfunction.

PD-specific assessments were performed by two senior neurologists (associate chief physicians or above) who were not involved in the surgical procedures. In case of disagreement between the two raters, a third neurologist (chief physician) made the final determination.

### 2.5 Follow-up

Follow-up was conducted by trained study personnel according to a prespecified protocol. All participants were followed from the date of surgery to 12 months postoperatively (±2 weeks allowed) using a combination of telephone interviews, scheduled outpatient visits, and WeChat-based contact when appropriate. Follow-up assessments included medication adjustments, postoperative complications and rehospitalization events, stimulation programming records, and PD-related scale evaluations. The primary endpoint was the Schwab and England Activities of Daily Living scale (S&E) assessed at 12 months in the medication-off state. To ensure consistency, scale assessments were performed whenever possible after withholding levodopa preparations for at least 12 h (i.e., during the medication-off period) following routine clinical practice. The initial stimulation amplitude at first programming was recorded (V); subsequent adjustments were individualized according to clinical response and adverse effects, and the typical amplitude range in routine practice was approximately 1.5–5.0 V.

To minimize information bias, outcome assessments were completed by neurologists and/or research staff who were not involved in the surgical procedures. When complete scale assessment could not be reliably performed via telephone or online contact, patients were preferentially scheduled for an in-person outpatient evaluation to ensure standardized measurements. For participants who could not be reached or declined follow-up, at least three contact attempts were made on different days and at different times; those without ascertainable outcome information were classified as lost to follow-up and were excluded from the final analysis. All follow-up data were recorded using standardized case report forms, independently checked by two investigators, and subject to periodic quality control; discrepant or uncertain information was verified by reviewing medical records and/or re-contacting the patient to ensure data completeness and accuracy. To protect patient privacy and data security, online communication platforms (including WeChat) were used only for scheduling follow-up and verifying information and were not used to transmit sensitive identifiable materials (e.g., national identification numbers or complete medical imaging records). All follow-up data were de-identified using study-specific codes and stored in access-restricted, password-protected and/or encrypted systems, accessible only to authorized study investigators.

### 2.6 Study design and statistical analysis

This study was a retrospective cohort study conducted at a single center. Screening was performed on patients diagnosed with Parkinson’s disease (PD) who had undergone subthalamic nucleus deep brain stimulation (STN-DBS) at the Department of Functional Neurosurgery, Brain Hospital of Liaocheng People’s Hospital between the dates of January 1, 2015 and August 1, 2024. Every single patient was scheduled to undergo a follow-up examination twelve months after the surgical procedure, with a window of approximately two weeks. The Schwab and England Activities of Daily Living scale (S&E) was the major outcome that was evaluated with the patient in the medication-off state after a period of twelve months. A good outcome was defined as a Standard Error (S&E) that was greater than 70, while a poor outcome was defined as an S&E that was less than or equal to 70. Patients who did not have any data that could be determined about their outcomes after a year were categorized as lost to follow-up and were not included in the final analysis.

The majority of the tasks, such as data preprocessing, descriptive analysis, comparisons between groups, and regression modeling, were carried out with the assistance of Python (version 3.11.1). In accordance with the conditions, continuous variables were summarized as the mean plus or minus the standard deviation or the median (interquartile range). In order to carry out comparisons, either the independent-samples t test or the Mann–Whitney U test was taken into consideration. In order to compare the categorical variables, we applied both the chi-square test (with continuity correction when indicated) and Fisher’s exact test. Both of these tests were utilized. We reported the categorical variables as counts, which are often known as percentages. In order to be considered statistically significant, the *p* values would need to have two sides that were lower than 0.05.

Candidates for the role of predictors were prespecified and extracted from three distinct domains: (1) general clinical aspects, (2) perioperative indicators, and (3) preoperative PD-specific assessments. These domains were chosen because they were considered to be the most relevant. In the first stage of the procedure, univariable logistic regression was performed on each candidate variable in order to identify independent predictors of poor results. This was done in order to find out what factors predicted poor outcomes. The factors that were deemed clinically significant and/or those that shown statistical significance in univariable analysis (often with a *p*-value less than 0.1) were added into a multivariable logistic regression model. The final set of variables that were retained in the multivariable model were utilized in the construction of a nomogram. Statistical significance and clinical interpretability were the two primary considerations that led to the selection of these predictors. The ‘rms’ package in R was used for nomogram visualization. R (version 4.5.2) was utilized for this specific endeavor.

For the purpose of determining the degree to which the model is capable of discrimination, the receiver operating characteristic (ROC) analysis was utilized. This investigation includes the area under the curve [AUC (C-index)], as well as the sensitivity and specificity at the optimal cutoff, which were determined by utilizing the Youden index during the calculation process. In order to measure optimism and acquire an optimism-corrected C-index along with confidence intervals of 95%, an internal validation was carried out. This was accomplished by applying bootstrap resampling with a total of one thousand repetitions. When evaluating the calibration, a bootstrap-corrected calibration plot was applied, and the Hosmer–Lemeshow goodness-of-fit test was utilized in order to further analyze the calibration. Both of these methods were utilized in order to evaluate the calibration. The decision curve analysis (DCA) was utilized in order to investigate the clinical utility. This analysis involved contrasting the “treat-all” and “treat-none” strategies with regard to the net benefit that was obtained across a range of threshold probabilities. The threshold probability interval that contained the net clinical benefit was subsequently reported when it had been determined. With the exception of few instructions that were altered, the analyses were carried out by using complete cases for the variables that were incorporated into the final model.

## 3 Result

### 3.1 Follow-up overview

Participants in the trial included 195 individuals who had been diagnosed with Parkinson’s disease (PD). These individuals were scheduled to undergo a postoperative follow-up after a period of 12 months, with a margin of error of ±2 weeks throughout the duration of the study. It was determined that there were 11 patients who were not followed up on during the follow-up period, which led to a follow-up completion rate of 94.4% for the follow-up. A number of different approaches were utilized in order to carry out the process of following up, including conducting interviews over the phone, attending outpatient appointments, and interacting through WeChat. A minimum of three attempts were made by the study team to communicate with each individual patient, each of which was conducted on a different day and at a different time. Patients for whom it was not possible to gather information regarding the outcome after a year were classified as “lost to follow-up” and were not included in the conclusions of the study. After everything was said and done, a total of 184 patients were incorporated into the statistical analysis. The patients who were included in this study were divided into two groups: 109 patients who were in the good-outcome group (medication-off Schwab and England Activities of Daily Living scale [S&E] > 70) and 75 patients who were in the poor-outcome group (medication-off S&E ≤ 70). It was possible to successfully determine the primary outcome for each and every patient who participated in the trial. Furthermore, the follow-up data were exhaustive and appropriate for the purpose of conducting additional comparisons between groups, as well as for the building and validation of the nomogram prediction model.

### 3.2 Comparison of general clinical characteristics

When compared to the group that had a favorable outcome, the group that had a poor outcome was older, had a later age at onset, and had a longer duration of disease (age: *Z* = −5.853, *p* < 0.001; age at onset: *Z* = −4.468, *p* < 0.001; disease duration: *t* = −2.593, *p* = 0.010). A number of other general clinical features, including comorbidities, education level, body mass index, and gender, were found to be comparable between the groups (all *p* > 0.05). The results are presented in Supplementary Table S2 in their entirety.

### 3.3 Comparison of perioperative indicators

When compared to the group that had a good outcome, the group that had a bad outcome had a longer duration of surgery (*Z* = −4.092, *p* < 0.001), a greater proportion of ASA grade ≥II (*χ*^2^ = 8.683, *p* = 0.003), and a higher incidence of postoperative electrolyte disturbance (*χ*^2^ = 5.220, *p* = 0.022). There was no significant difference between the groups in terms of other perioperative markers, such as the amount of time spent under anesthesia, blood loss, preoperative hemoglobin, preoperative albumin, or preoperative pneumonia (all *p* > 0.05). The results are presented in Supplementary Table S2 in their entirety. In addition, the initial stimulation amplitude at first programming was comparable between groups (good outcome: 2.51 ± 0.87 V; poor outcome: 2.48 ± 0.86 V); amplitudes were subsequently titrated stepwise during follow-up according to symptoms and side effects, typically within the range of 1.5–5.0 V in routine practice. Lead placement quality-control based on postoperative thin-slice CT fused with preoperative MRI showed consistently satisfactory targeting, with deviations typically within 2 mm and no revisions for malposition. A concise summary of lead-placement verification and stimulation parameters is provided in Supplementary Table S3.

### 3.4 Comparison of preoperative PD-specific indicators

In comparison to the group that had a good outcome, the group that had a poor outcome had higher preoperative UPDRS-II scores (*Z* = −10.022, *p* < 0.001) and a higher H&Y stage (*Z* = −10.974, *p* < 0.001). As an additional point of interest, the group with poor outcomes demonstrated lower MMSE scores (*Z* = 6.362, *p* < 0.001). Moreover, the UPDRS-III and the PDQ-39 surveys both revealed differences (*t* = 2.174, *p* = 0.031 for the UPDRS-III survey and *t* = 3.482, *p* = 0.001 for the PDQ-39 survey). These findings were based on statistical analysis. There was no significant difference between the groups in terms of other preoperative PD-related variables, such as BAI, BDI, NMSS, PDSS, and LEDD (all *p* > 0.05). This conclusion was reached after analyzing the data. Supplementary Table S2, which contains the specifics of the findings, is shown here.

### 3.5 Logistic regression analysis for predictors of poor outcome after STN-DBS

Poor postoperative outcome following STN-DBS was the dependent variable. Variables showing significant between-group differences were entered into logistic regression models, including general clinical characteristics, perioperative indicators, and Parkinson’s disease–specific preoperative assessments. In univariable analyses, age, Mini-Mental State Examination (MMSE), postoperative electrolyte disorder, operation time, Unified Parkinson’s Disease Rating Scale part III (UPDRS-III), disease duration, and ASA grade (≥II) were associated with poor outcome (all *p* < 0.05). In multivariable analysis, older age (OR = 1.08, 95% CI 1.03–1.14, *p* = 0.001), lower MMSE (OR = 0.66, 95% CI 0.56–0.79, *p* < 0.001), and postoperative electrolyte disorder (OR = 2.97, 95% CI 1.28–6.91, *p* = 0.011) remained independent predictors. The detailed regression results are presented in Table 1. Key predictors retained in the final nomogram are summarized in Table 2.

**Table 1:** Logistic regression analysis (with Wald Z).

Variables	Univariate P	Univariate Wald Z	Multivariate OR (95% CI)	Multivariate P	Multivariate Wald Z
Age	<0.001	Z = 5.384	1.08 (1.03–1.14)	0.001	Z = 3.282
MMSE	<0.001	Z = -5.766	0.66 (0.56–0.79)	<0.001	Z = -4.734
Postop electrolyte disorder	0.015	Z = 2.426	2.97 (1.28–6.91)	0.011	Z = 2.533
Surgery time	<0.001	Z = 3.863	1.00 (1.00–1.01)	0.273	Z = 1.096
UPDRS-III	0.033	Z = -2.130	0.92 (0.80–1.07)	0.279	Z = -1.083
Disease duration	0.012	Z = 2.522	0.98 (0.86–1.12)	0.769	Z = -0.293
ASA Grade (≥II vs. I)	0.002	Z = 3.067	1.16 (0.48–2.79)	0.738	Z = 0.334

**Table 2:** Comparison of key predictors between outcome groups.

Predictor	Good outcome (*n* = 109)	Poor outcome (*n* = 75)	*p* value
Age (years), median (IQR)	57.10 (52.89, 64.25)	66.39 (57.60, 72.11)	<0.001
MMSE, median (IQR)	26.57 (25.60, 28.20)	23.40 (20.97, 26.03)	<0.001
Postoperative electrolyte disorder, n (%)	24 (22.0%)	29 (38.7%)	0.022

MMSE, Mini-Mental State Examination; IQR, interquartile range.

### 3.6 Construction and application of the nomogram model

**Figure 1:**
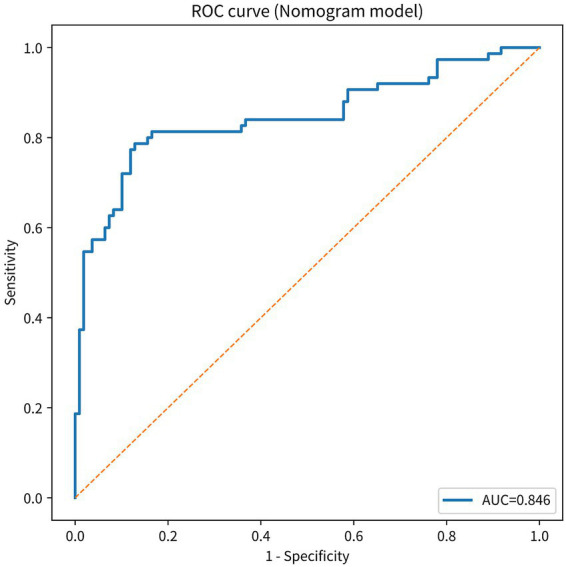
Nomogram for predicting poor 1-year functional outcome after STN-DBS in Parkinson’s disease. The nomogram was developed from multivariable logistic regression and includes age (years), preoperative Mini-Mental State Examination (MMSE) score, and postoperative electrolyte disorder during hospitalization. For each patient, locate the value of each predictor on its axis, draw a vertical line to the “Points” scale, and sum the points to obtain “Total Points.” Total points are then mapped to the predicted probability of poor outcome on the “Risk of Poor Prognosis” axis. In this study, poor outcome was defined as a medication-off Schwab and England Activities of Daily Living scale (S&E) score ≤70 at 12 months. STN-DBS, subthalamic nucleus deep brain stimulation; MMSE, Mini-Mental State Examination; S&E, Schwab and England Activities of Daily Living scale.

Estimating the individual risk of poor outcome following STN-DBS was accomplished by the development of a nomogram that was based on the independent predictors that were discovered through multivariable logistic regression. Age (age_years), preoperative MMSE score (mmse), and postoperative electrolyte condition during hospitalization (postop_electrolyte_disorder) were all factors that were integrated into the final nomogram. The nomogram is organized in such a way that each predictor corresponds to a point scale. Points are allocated by identifying the patient’s value on each predictor axis and then projecting upward to the “Points” scale. After adding together all of the points for each predictor, the “Total Points” are obtained. These “Total Points” are then mapped to the “Risk of Poor Prognosis” axis, which results in the individualized predicted probability of a negative outcome (Figure 1).

### 3.7 Discrimination of the nomogram model: ROC analysis

**Figure 2:**
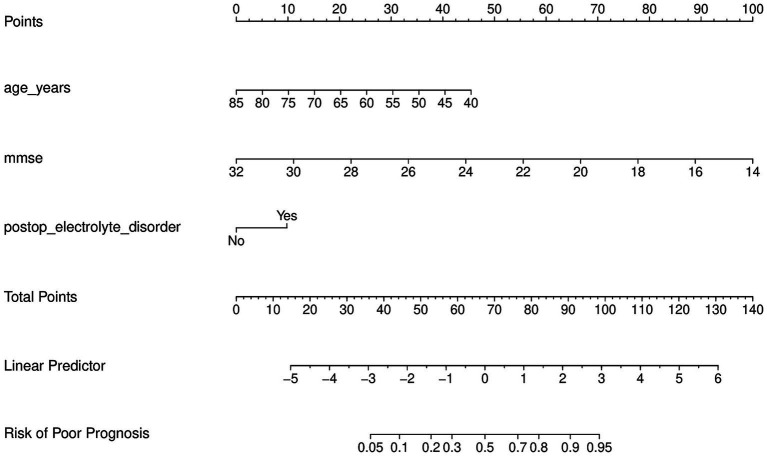
Receiver operating characteristic (ROC) curve of the nomogram model. The ROC curve illustrates discrimination of the prediction model for poor 1-year functional outcome after STN-DBS. The area under the curve (AUC) is 0.846. ROC, receiver operating characteristic; AUC, area under the curve; STN-DBS, subthalamic nucleus deep brain stimulation.

A significant discrimination was indicated by the ROC analysis, which showed that the nomogram model that included age (age_years), MMSE, and postoperative electrolyte disorder (postop_electrolyte_disorder) had a strong discrimination. The AUC (C-index) for this model was 0.846 (bootstrap 95% confidence interval 0.781–0.905). The evidence for this can be found in Figure 2. For example, the utilization of the Youden index resulted in the acquisition of a sensitivity of 0.787 and a specificity of 0.872. This, in turn, led to the conclusion that the suitable cutoff value is 0.501. Additionally, the positive predictive value (PPV) was 0.808, and the negative predictive value (NPV) was 0.856. Both of these values are quite significant. From a general standpoint, the outcomes were fairly positive.

### 3.8 Calibration of the nomogram model: bootstrap calibration and goodness-of-fit

**Figure 3:**
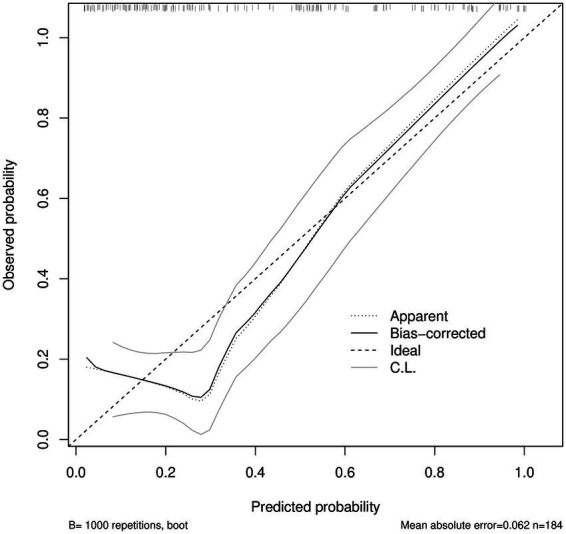
Bootstrap-corrected calibration plot of the nomogram model. Calibration of predicted probabilities against observed outcomes using bootstrap resampling (*B* = 1,000; *n* = 184). The dotted line indicates the apparent calibration, the solid line indicates the bias-corrected calibration, and the dashed line represents the ideal (45°) line. Grey curves denote confidence limits (C. L.). Mean absolute error (MAE) is 0.062. MAE, mean absolute error.

Furthermore, as can be seen in Figure 3, the bootstrap calibration plot (*B* = 1,000; *n* = 184) demonstrated that the predicted probabilities and the observed probabilities were, for the most part, in accord with one another. In addition, the bias-corrected curve was situated in close proximity to the ideal line, suggesting generally acceptable calibration. However, the Hosmer–Lemeshow test was statistically significant (*p* = 0.031), indicating some deviation from perfect calibration; given the known sensitivity of the Hosmer–Lemeshow test to sample size and grouping, we primarily relied on the bootstrap-corrected calibration curve and calibration error metrics to judge overall calibration. The goodness-of-fit test conducted by Hosmer and Lemeshow yielded a result of *χ*^2^ = 16.893 (df = 8), accompanied with a *p*-value of 0.031.

### 3.9 Calibration and clinical utility of the nomogram: calibration plot and decision curve analysis

**Figure 4:**
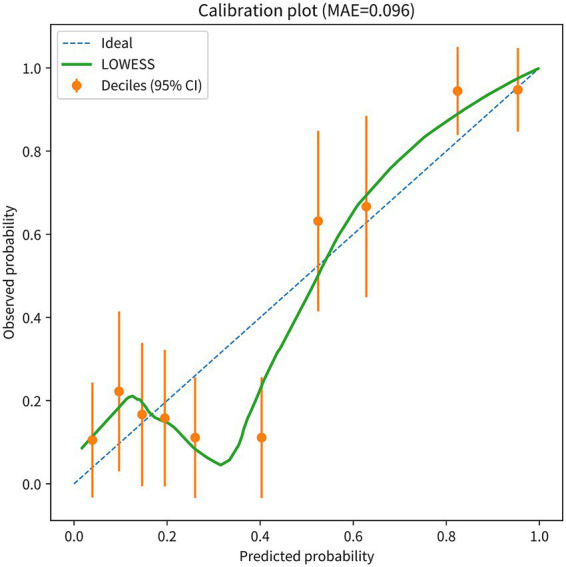
Calibration performance of the nomogram model. Calibration plot showing agreement between predicted and observed probabilities of poor outcome (*n* = 184). The LOWESS curve summarizes calibration across the probability range; points represent deciles of predicted risk with 95% confidence intervals. The ideal reference line is shown for comparison. Mean absolute error (MAE) is 0.096. LOWESS, locally weighted scatterplot smoothing; MAE, mean absolute error.

The bootstrap-corrected calibration plot (*B* = 1,000; Figure 3) showed a mean absolute error (MAE) of 0.062, indicating general agreement between predicted and observed probabilities. In the decile-based calibration summary (Figure 4), the MAE was 0.096. We therefore report both the plot-based calibration assessment and the Hosmer–Lemeshow test results for transparency.

**Figure 5:**
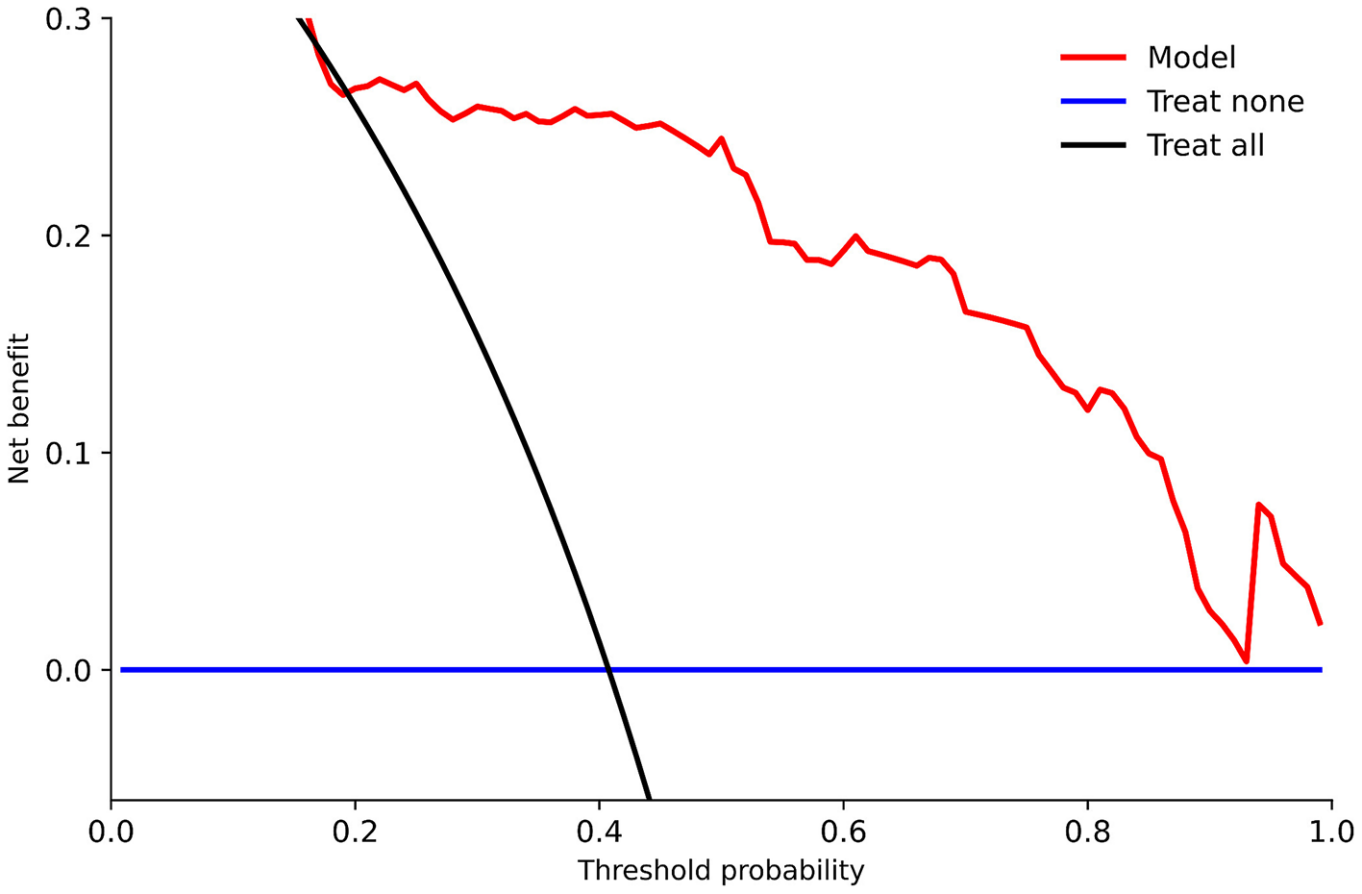
Decision curve analysis (DCA) for the nomogram model. Decision curve analysis comparing the net benefit of the nomogram model with “treat-all” and “treat-none” strategies across a range of threshold probabilities for poor outcome. A higher net benefit indicates greater potential clinical utility for risk-based decision-making. DCA, decision curve analysis.

Based on the findings of a decision curve analysis (DCA), it was determined that the nomogram was capable of producing a greater net benefit than both the “treat-all” and “treat-none” strategies across a broad threshold probability range of 0.20–0.99. In addition, the decision curve showed two narrower threshold-probability windows (0.02–0.04 and 0.14–0.16) in which the nomogram provided a modest incremental net benefit compared with the default strategies. According to the scenario in which the probability barrier was established at 0.01, the highest possible net benefit was 0.402. The net advantage of the model was 0.268 when the Pt value was 0.20, 0.259 when the Pt value was 0.30, and 0.245 when the Pt value was 0.50 (Figure 5; Supplementary Table S1). To facilitate interpretation, we also summarized net benefit values at commonly used threshold probabilities in Supplementary Table S1.

## 4 Discussion

### 4.1 Principal findings and clinical implications: functional outcome–oriented individualized risk assessment

Through the utilization of a patient-centered endpoint, we were able to create a tailored prediction model within this real-world, single-center cohort. Using the Schwab and England Activities of Daily Living scale (S&E), this endpoint was analyzed to determine whether or not the individual had achieved functional independence after a year. It was discovered that there was a significant amount of variability in functional recovery at the one-year follow-up, which highlights the frequent clinical conundrum of “similar surgery, different outcomes.” In spite of the fact that STN-DBS is generally useful for the treatment of symptoms in Parkinson’s disease (PD), it was discovered that functional recovery was highly variable. Our team developed a nomogram that is not only practical at the bedside but also as simple as possible. The information that is readily available is transformed into a tailored probability of a terrible outcome by means of this nomogram. This was achieved by doing a rigorous examination of general clinical parameters, perioperative symptoms, and preoperative assessments that were unique to Parkinson’s disease. This technology has the potential to provide assistance in a number of different areas, including preoperative counseling, perioperative risk assessment, and the rational distribution of resources for follow-up and rehabilitation. Particularly interesting is the fact that the model preserved three important indicators: age, preoperative MMSE, and postoperative electrolyte issue. These are among the most relevant predictors. In multivariable analysis, all three characteristics continued to be independently connected with a bad result (see Table 1 for more information concerning this).

### 4.2 Interpretation of key predictors: age, cognition, and an “actionable” perioperative factor

Older age was associated with a higher likelihood of poor 1-year functional outcome, whereas higher MMSE scores were protective (21, 22). Cognitive status may influence early postoperative participation in rehabilitation, the efficiency of DBS programming optimization, medication adherence, and ultimately the ability to regain functional independence (23–26).

Importantly, postoperative electrolyte disturbance during hospitalization emerged as an independent predictor (OR 2.97, *p* = 0.011). Although electrolyte abnormalities are often transient, they can plausibly influence longer-term outcomes through several pathways. First, electrolyte imbalance may precipitate acute brain dysfunction (e.g., delirium) (26–28), and delirium in turn is associated with prolonged recovery, reduced engagement in rehabilitation, and persistent cognitive/functional decline in vulnerable older patients (29). In PD patients undergoing DBS, postoperative delirium is increasingly recognized, with recent evidence highlighting older age and preoperative cognitive impairment as key risk factors (30, 31). Second, electrolyte disturbance frequently reflects systemic stress and physiological vulnerability (e.g., autonomic dysfunction, inadequate oral intake, infection/inflammation, renal dysfunction, or medication-related effects), which may increase the risk of perioperative complications and delay the stabilization needed for timely postoperative programming and rehabilitation. Third, in PD specifically, homeostatic instability can exacerbate non-motor symptoms (sleep disturbance, orthostatic intolerance) and worsen day-to-day function, thereby reducing the likelihood of achieving an S&E > 70 at 12 months.

Taken together, we interpret postoperative electrolyte disturbance as a clinically actionable marker of perioperative instability that may adversely affect the early recovery trajectory and, indirectly, 1-year functional independence. This also highlights a potentially modifiable target: proactive monitoring and early correction of fluid–electrolyte imbalance, along with optimization of nutrition and prevention/early treatment of intercurrent complications, may help create more favorable conditions for recovery after STN-DBS—particularly in older patients and those with lower baseline cognition.

### 4.3 Model performance and practical utility: integrated evidence from discrimination, calibration, and decision-curve analysis

Furthermore, calibration assessment showed general agreement between predicted and observed risks. The bootstrap-corrected calibration curve was close to the ideal line, and the mean absolute error (MAE) was 0.062 (Figure 3) and 0.096 in the decile-based summary (Figure 4). Notably, the Hosmer–Lemeshow test yielded *p* = 0.031, suggesting a statistically detectable departure from perfect fit; because this test can be overly sensitive to sample size and the choice of grouping, we interpret it alongside the bootstrap-corrected calibration plots and MAE to conclude that calibration is generally acceptable, while acknowledging potential minor miscalibration.

### 4.4 Limitations and future directions: external validation, model extension, and clinical translation

There are a lot of constraints that need to be handled on the situation. The first thing to note is that this is a retrospective study that was carried out at a single location. In spite of the fact that bootstrap internal validation was performed, it is not feasible to completely exclude the possibilities of selection bias and information bias. It is essential to obtain external validation in multicenter cohorts in order to ensure that the model can be generalized without any problems. To achieve the best possible results, this validation should be carried out across a wide range of surgical teams and follow-up settings. Second, the medication-off S&E scale was used to define functional result after 1 year. This type of measure is clinically meaningful, but it may still be influenced by contextual factors such as social support, the intensity of rehabilitation, and the diversity in follow-up modalities. In subsequent research, it may be possible to incorporate more patient-reported outcomes as well as more objective functional measurements in order to increase the robustness of the phenotype. Third, the model does not incorporate potentially informative dynamic variables, such as imaging-based targeting accuracy, stimulation parameters, longitudinal programming trajectories, or detailed postoperative medication adjustments. This is despite the fact that the model was purposefully kept as simple as possible in order to make bedside implementation easier. Enhanced predictive accuracy and a better understanding of inter-individual variability could be achieved through the investigation of hybrid frameworks in the future. These frameworks would combine “baseline risk” with time-varying perioperative and programming information. In conclusion, it is necessary to have prospective external validation that includes predetermined reporting of calibration and clinical utility, in addition to practical implementation paths (for example, risk-stratified perioperative monitoring methods). The creation of an online calculator or the incorporation of the model into clinical information systems may be additional ways to facilitate the adoption of the model in the real world and the transformation of the research model into a clinical tool that can be utilized.

## 5 Conclusion

In this study, a customized nomogram was generated and assessed in order to predict the functional result of patients with Parkinson’s disease who were undergoing STN-DBS over a period of 1 year (medication-off state evaluation). After conducting a multivariable analysis, it was determined that the following factors were independent predictors of poor outcome: older age, poorer preoperative MMSE score, and postoperative electrolyte imbalance during hospitalization. The nomogram that was produced as a result revealed good discrimination and calibration, and it indicated potential net clinical benefit on decision curve analysis. This lends support to the utilization of the nomogram for perioperative risk assessment, shared decision-making, and tailored follow-up planning. In light of the fact that the design was a retrospective one, it is necessary to conduct external validation in multicenter cohorts and prospective studies before implementing the study on a large scale in clinical settings.

## Data Availability

The raw data supporting the conclusions of this article will be made available by the authors, without undue reservation.
