# Supplementary material for: A parsimonious nomogram for individualized prediction of 1-year functional outcome after STN-DBS in Parkinson’s disease: a single-center retrospective study
Source: Front Neurol. 2026 Feb 20;17:1779907. doi: 10.3389/fneur.2026.1779907 (PMC12962900; doi:10.3389/fneur.2026.1779907)
Supplement: Supplementary file 1 [file Supplementary_file_1.docx]

**Supplementary Methods S1 Detailed STN-DBS surgical procedure**

The following text provides the detailed surgical procedure that was moved from the main Methods section.

At twelve hours prior to surgery, all of the patients stopped using oral levodopa preparations. While the patient was under the influence of local anesthesia, a stereotactic head base was applied with reference to the line that was parallel to the anterior commissure–posterior commissure (AC–PC) straight line. A CT scan was performed, and the images were imported into the Framlink system. Based on the preoperative surgical plan, bilateral subthalamic nucleus (STN) targets were defined, and the patient was transferred to the operating room.

The patient was placed in the supine position with the head elevated by 20°. After routine skin preparation and sterile draping, the stereotactic frame was mounted. Under local anesthesia, implantation of the left electrode was performed first. A burr-hole entry point was selected 3.5 cm lateral to the left frontal midline. A 4-cm arcuate incision was made at the level of the coronal suture, the scalp was retracted, and a burr hole was created using a hand drill. The dura mater was coagulated and opened in a cruciate fashion. A guiding device was installed, and a microelectrode was advanced. When typical STN signals were identified on microelectrode recording, the target was documented and the definitive stimulation electrode was implanted. Intraoperative test stimulation was performed; after increasing the stimulation voltage without obvious adverse effects, the electrode was detached from the guiding device and secured at the burr hole, with the contacts protected by a cap. The right electrode was implanted using the same technique. The right lead was then tunneled subcutaneously to the left side. Both leads were temporarily placed in the subcutaneous tunnel at the left incision site. The bilateral cranial incisions were closed in layers and dressed.

After completion of the above steps, the stereotactic frame base was removed. The patient was repositioned supine, and general anesthesia with endotracheal intubation was induced. A 5-cm transverse incision was made approximately one fingerbreadth below the left clavicle, and a 5 cm × 5 cm pocket was created between the deep pectoral fascia and the pectoralis major muscle, temporarily packed with gauze. A 3-cm vertical incision was made 3 cm superior and posterior to the left auricle, and subperiosteal dissection was performed. A subcutaneous tunnel was created with a probe, and the pulse generator was advanced through the tunnel to the retroauricular incision. The extracranial portion of the DBS leads was retrieved, and the lead ends were connected to the extension cable at the retroauricular incision; the connection was protected with a cap and secured to the skull. The pulse generator was implanted and fixed within the subclavicular pocket, and all incisions were closed in layers.
