# Supplementary material for: A parsimonious nomogram for individualized prediction of 1-year functional outcome after STN-DBS in Parkinson’s disease: a single-center retrospective study
Source: Front Neurol. 2026 Feb 20;17:1779907. doi: 10.3389/fneur.2026.1779907 (PMC12962900; doi:10.3389/fneur.2026.1779907)
Supplement: Supplementary file 2 [file Table_1.docx]

**Supplementary Table1 Key net benefit values at common thresholds**

| Threshold | NB_model | NB_treat_all | NB_treat_none | NB_model_minus_all | NB_model_minus_none |
| --- | --- | --- | --- | --- | --- |
| 0.050 | 0.369 | 0.376 | 0.000 | -0.007 | 0.369 |
| 0.100 | 0.329 | 0.342 | 0.000 | -0.013 | 0.329 |
| 0.150 | 0.304 | 0.303 | 0.000 | 0.001 | 0.304 |
| 0.200 | 0.268 | 0.260 | 0.000 | 0.008 | 0.268 |
| 0.300 | 0.259 | 0.154 | 0.000 | 0.106 | 0.259 |
| 0.500 | 0.245 | -0.185 | 0.000 | 0.429 | 0.245 |
