# Supplementary material for: A parsimonious nomogram for individualized prediction of 1-year functional outcome after STN-DBS in Parkinson’s disease: a single-center retrospective study
Source: Front Neurol. 2026 Feb 20;17:1779907. doi: 10.3389/fneur.2026.1779907 (PMC12962900; doi:10.3389/fneur.2026.1779907)
Supplement: Supplementary file 3 [file Table_2.docx]

**Supplementary Table S2. Baseline comparison between the good- and poor-outcome groups**

| **Variables** | **Good Outcome (n=109)** | **Poor Outcome (n=75)** | **Test** | **Statistic** | **P-value** |
| --- | --- | --- | --- | --- | --- |
| **A. General clinical data** | | | | | |
| Age (years) | 57.10 (52.89, 64.25) | 66.39 (57.60, 72.11) | Mann–Whitney U | U=2010, Z=-5.853 | <0.001 |
| Gender (Male, n(%)) | 66 (60.6%) | 34 (45.3%) | Chi-square (Yates) | χ²=3.556 | 0.059 |
| BMI (kg/m²) | 28.49 (24.26, 30.18) | 27.82 (24.89, 30.31) | Mann–Whitney U | U=4256, Z=0.473 | 0.636 |
| Disease Duration (years) | 7.91 ± 3.36 | 9.22 ± 3.40 | Student t-test | t=-2.593 | 0.010 |
| Age of Onset (years) | 50.67 (44.50, 53.62) | 56.03 (50.00, 61.15) | Mann–Whitney U | U=2502, Z=-4.468 | <0.001 |
| Education (≥HighSchool, n(%)) | 27 (24.8%) | 13 (17.3%) | Chi-square (Yates) | χ²=1.040 | 0.308 |
| Hypertension (n(%)) | 69 (63.3%) | 41 (54.7%) | Chi-square (Yates) | χ²=1.042 | 0.307 |
| Diabetes (n(%)) | 39 (35.8%) | 33 (44.0%) | Chi-square (Yates) | χ²=0.939 | 0.333 |
| CHD (n(%)) | 47 (43.1%) | 38 (50.7%) | Chi-square (Yates) | χ²=0.737 | 0.391 |
| Stroke History (n(%)) | 45 (41.3%) | 31 (41.3%) | Chi-square (Yates) | χ²=0.000 | 1.000 |
| **B. Perioperative indicators** | | | | | |
| Surgery Time (min) | 108.26 (77.17, 169.90) | 172.21 (122.60, 232.09) | Mann–Whitney U | U=2635, Z=-4.092 | <0.001 |
| Anesthesia Time (min) | 166.07 (122.32, 229.60) | 172.74 (138.07, 232.32) | Mann–Whitney U | U=3665, Z=-1.190 | 0.234 |
| Blood Loss (ml) | 142.55 (120.96, 165.10) | 138.20 (122.40, 167.23) | Mann–Whitney U | U=4214, Z=0.356 | 0.722 |
| Preop Hb (g/L) | 127.07 (118.40, 136.30) | 130.57 (117.08, 140.15) | Mann–Whitney U | U=3666, Z=-1.186 | 0.236 |
| Preop Albumin (g/L) | 38.60 (36.91, 40.50) | 39.50 (36.29, 42.11) | Mann–Whitney U | U=3832, Z=-0.721 | 0.471 |
| ASA Grade (≥II, n(%)) | 36 (33.0%) | 42 (56.0%) | Chi-square (Yates) | χ²=8.683 | 0.003 |
| Preop Pneumonia (n(%)) | 26 (23.9%) | 16 (21.3%) | Chi-square (Yates) | χ²=0.049 | 0.825 |
| Postop Electrolyte Disorder (n(%)) | 24 (22.0%) | 29 (38.7%) | Chi-square (Yates) | χ²=5.220 | 0.022 |
| **C. Preoperative specialist indicators** | | | | | |
| UPDRS-II | 29.41 (26.30, 30.43) | 37.30 (32.63, 40.59) | Mann–Whitney U | U=530, Z=-10.022 | <0.001 |
| UPDRS-III | 43.19 ± 2.95 | 42.26 ± 2.72 | Student t-test | t=2.174 | 0.031 |
| H&Y Stage | 2.02 (1.50, 2.50) | 3.50 (3.01, 3.99) | Mann–Whitney U | U=198, Z=-10.974 | <0.001 |
| MMSE | 26.57 (25.60, 28.20) | 23.40 (20.97, 26.03) | Mann–Whitney U | U=6346, Z=6.362 | <0.001 |
| BAI | 14.10 (11.63, 15.80) | 14.70 (11.51, 16.07) | Mann–Whitney U | U=3891, Z=-0.554 | 0.580 |
| BDI | 10.30 ± 3.00 | 10.86 ± 3.36 | Student t-test | t=-1.174 | 0.242 |
| NMSS | 89.78 ± 34.52 | 97.68 ± 34.09 | Student t-test | t=-1.532 | 0.127 |
| PDSS | 90.63 ± 29.97 | 89.82 ± 30.05 | Student t-test | t=0.182 | 0.856 |
| PDQ-39 | 80.80 ± 24.09 | 68.72 ± 21.64 | Student t-test | t=3.482 | 0.001 |
| LEDD (mg/day) | 448.07 ± 88.07 | 460.90 ± 88.16 | Student t-test | t=-0.971 | 0.333 |
