# Supplementary material for: A parsimonious nomogram for individualized prediction of 1-year functional outcome after STN-DBS in Parkinson’s disease: a single-center retrospective study
Source: Front Neurol. 2026 Feb 20;17:1779907. doi: 10.3389/fneur.2026.1779907 (PMC12962900; doi:10.3389/fneur.2026.1779907)
Supplement: Supplementary file 4 [file Table_3.docx]

**Supplementary Table S3. Lead placement accuracy and stimulation parameters by outcome group**

| **Variables** | **Good outcome (n=109)** | **Poor outcome (n=75)** | **Test** | **Statistic** | **P-value** |
| --- | --- | --- | --- | --- | --- |
| Initial stimulation amplitude at first programming (V), mean ± SD | 2.51 ± 0.87 | 2.48 ± 0.86 | Welch’s t-test | t=0.231, df=160.4 | 0.817 |
| Typical stimulation amplitude adjustment range during follow-up (V) | 1.5–5.0 | 1.5–5.0 | — | — | — |
| Lead placement accuracy (postop thin-slice CT fused with preop MRI) | Deviation typically ≤2 mm; no revision for malposition | Deviation typically ≤2 mm; no revision for malposition | — | — | — |
